# Structure activity relationships in metal–organic framework catalysts for the continuous flow synthesis of propylene carbonate from CO_2_ and propylene oxide†

**DOI:** 10.1039/c7ra13245j

**Published:** 2018-01-09

**Authors:** Bryant R. James, Jake A. Boissonnault, Antek G. Wong-Foy, Adam J. Matzger, Melanie S. Sanford

**Affiliations:** Department of Chemistry, University of Michigan 930 North University Avenue Ann Arbor MI 48109 USA mssanfor@umich.edu; Macromolecular Science & Engineering, College of Engineering, University of Michigan 930 North University Avenue Ann Arbor MI 48109 USA

## Abstract

This paper describes the systematic study of metal–organic framework (MOF) catalysts for the reaction of propylene oxide (PO) with carbon dioxide (CO_2_) to generate propylene carbonate (PC). These studies began with the evaluation of MIL-101(Cr) as catalyst in a flow reactor. Under the developed flow conditions, MIL-101(Cr) was found to effectively catalyze PO carbonation in the absence of a halide co-catalyst. A systematic study of catalyst performance was then undertaken as a function of MOF synthesis technique, activation conditions, metal center, and node architecture. Ultimately, these investigations led to the identification of MIL-100(Sc) as a new, active, and stable catalyst for PO carbonation.

## Abstract 2

Studies of MOF catalysts under continuous flow have led to the discovery of a new catalyst for carbonation of propylene oxide.

## Graphical abstract

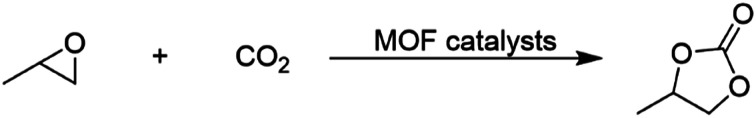

## Introduction

Metal–organic frameworks (MOFs) have been widely studied as catalysts for a variety of transformations.^1^ MOF-based catalysts combine well-defined, site-isolated metal active sites in structurally well-defined and recyclable scaffolds. In addition, the secondary and tertiary structure of MOFs can be systematically varied *via* modification at the organic linker and metal nodes of these structures.^2,3^ Thus, unlike most traditional heterogeneous catalysts, the active sites in MOFs can be rationally tuned to generate catalysts that are optimized for a specific reaction.^4^ The work described herein leverages the tunability of MOFs for the systematic study of catalysts for the reaction of carbon dioxide with propylene oxide to generate propylene carbonate.

**Scheme 1:**
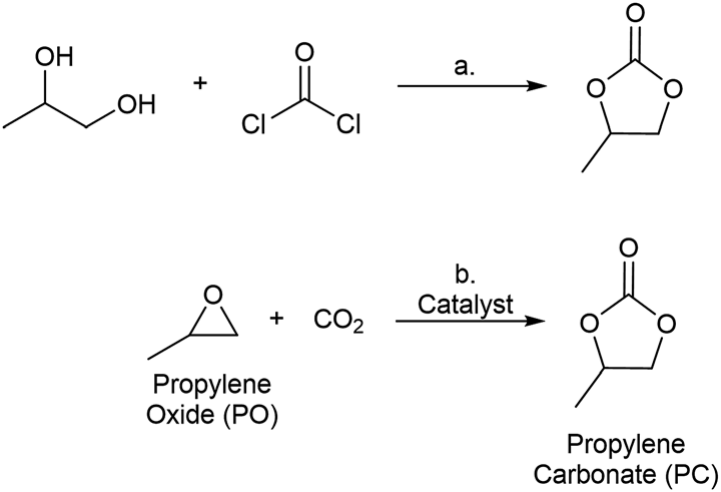
(a) Phosgene/diol route to propylene carbonate; (b) CO_2_/epoxide route to propylene carbonate.

Cyclic carbonates are commodity chemicals that are widely used as solvents for Li-ion batteries [*e.g.*, propylene carbonate (PC)] as well as monomers for polycarbonate synthesis.^5^ Cyclic carbonates are commonly prepared by the reaction of phosgene with the corresponding diol (Scheme 1a).^6,7^ An attractive alternative synthesis involves the reaction of epoxides [*e.g.*, propylene oxide (PO)] with CO_2_ to yield cyclic carbonate products (Scheme 1b).^8^ This transformation offers the advantages of high atom economy and the use of inexpensive and relatively non-toxic reagents.^9^ As such, a wide variety of both homogeneous and heterogeneous catalysts have been developed for epoxide carbonation, including single site metal complexes,^10–12^ metalloporphyrins,^13^ zeolites,^14,15^ ionic liquids,^16,17^ and MOFs.^18–23^ Despite a number of reports of MOF-based catalysts for PO carbonation, there are few examples of systematic studies of the impact of MOF structure on catalytic performance for this transformation.^24^

The majority of previous studies on MOF-catalysed PO carbonation have been performed in batch reactors.^23,25^ We reasoned that a flow configuration would be better suited to systematic investigations, as it would enable continuous analysis of the reaction profile. This paper demonstrates the evaluation of different MOF catalysts for PO carbonation, using the known catalyst MIL-101(Cr) as a starting point. Systematic variation of the synthesis technique, activation conditions, metal node, and organic linker were conducted in order to determine the key features necessary for catalysis and to optimize catalyst performance. These studies ultimately led to the identification of co-catalyst-free conditions for MOF-catalyzed PO carbonation and enabled the identification of MIL-100(Sc) as an improved catalyst for PO carbonation.

## Experimental

### Synthesis and characterization of catalysts

MIL-101(Cr),^26^ MIL-101(Fe),^27^ MIL-101(Sc),^28^ MIL-100(Sc),^28^ MIL-88D(Sc),^28^ and MIL-66(Sc)^28^ were all prepared according to reported procedures. All reagents were obtained from either Fisher or Sigma-Aldrich and used without further purification, with the exception of *N*,*N*′-dimethylformamide (DMF) (which was dried over 4 Å molecular sieves) and *N*,*N*′-diethylformamide (DEF) (which was purified by stirring over activated charcoal followed by filtration through silica gel).

Powder X-ray Diffraction (PXRD) data were recorded at room temperature on a Bruker AXS D8 Advance powder diffractometer at 40 kV, 40 mA with a CuKα source (*λ* = 1.5406 Å) between 3 and 30° 2*θ* with a scan speed of 0.1 s per step and a step size of 0.04. Samples were measured on a glass microscope slide in an aluminum holder. All powder patterns were taken in a mixture (1 : 3 or 1 : 1) of MOF to diatomaceous earth. The diatomaceous earth is visible as a sharp peak at 22° 2*θ*.

Temperature Programmed Desorption (TPD) data were collected on a Micromeritics ASAP 2920, using a quartz reactor with a quartz wool bed according to the following procedure. The line was purged with He for 15 min (20 mL min^−1^). NH_3_ was passed through the sample for 180 min (20 mL min^−1^) at 40 °C in order to saturate all acidic sites with NH_3_. The gas was switched to He, and He was passed over the sample for 30 min at 40 °C (30 mL min^−1^) in order to remove physisorbed NH_3_. The temperature was then ramped to 350 °C (5 °C min^−1^) to desorb the chemisorbed NH_3_, and the desorbed NH_3_ was detected *via* mass spectrometry.

### Typical procedure for catalytic testing

The catalyst and Fisher lab-grade diatomaceous earth were combined in either a 1 : 1 w/w ratio or a 1 : 3 w/w ratio. As a control, the diatomaceous earth was evaluated for reactivity in this reaction, and under the standard conditions it afforded <1% yield of PC. The mixture was transferred to a mortar and pestle and ground until visibly homogeneous. The mixture was packed between two glass wool plugs in a 1/4 inch OD, 1/20-inch wall thickness glass tube. The column was installed into the flow system, and both gas and stock solution streams were started simultaneously. CO_2_ flow rates were varied from 1–4 sccm min^−1^, and stock flow rates were varied from 0.25–0.5 mL min^−1^. A second column in series was designed into the system for larger quantities of catalyst. The second column was packed with glass wool when smaller quantities of catalyst were used. The system was allowed to run until reaching the desired system pressure (between 1 and 10 bar) before heating was started. Aliquots were collected every 30 to 60 min and analyzed by GC-FID on a Shimadzu GC-17A. Mesitylene was included in the feed as internal standard.

## Results and discussion

### Development of flow reaction conditions

MIL-101(Cr) was selected for initial study based on literature precedent that this material catalyzes PO carbonation in batch reactors in the presence of tetrabutylammonium bromide (TBABr) as a co-catalyst.^29,30^ In addition, this MOF is thermally robust, highly porous, and tunable at both the metal node and organic linker. As mentioned above, previous reports utilizing MIL-101(Cr) for PO carbonation involved catalysis conducted in batch reactors. We sought to translate this transformation to a flow reactor system in order to increase throughput as well as to facilitate continuous monitoring of the catalyst.

**Fig. 1:**
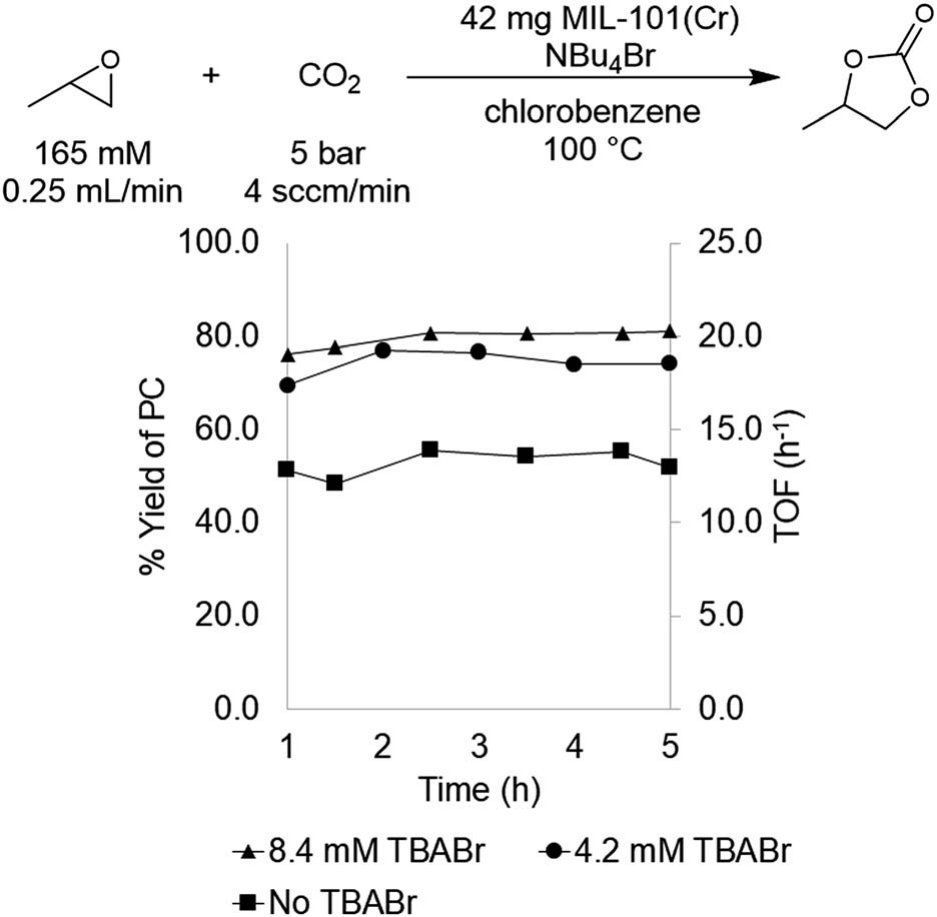
Effect of TBABr co-catalyst on PO carbonation catalyzed by MIL-101(Cr).

Initial flow reaction conditions were selected to closely mimic those used in batch.^25^ The flow reactions were conducted at 100 °C and 5 bar of system pressure with a CO_2_ flow rate of 4.0 sccm min^−1^. The MOF catalyst was a fixed bed of 42 mg of MIL-101(Cr) dispersed in 42 mg of diatomaceous earth. A stock solution of 165 mM propylene oxide and 8.4 mM tetrabutylammonium bromide (TBABr) co-catalyst in chlorobenzene was used to deliver both substrate and co-catalyst at a rate of 0.25 mL min^−1^. At steady state operation (established after approximately 1 h), these conditions afforded propylene carbonate with a TOF of 20 h^−1^ (Fig. 1). This corresponds to 0.033 mmol min^−1^ of propylene oxide produced in a single pass, equivalent to an 80 ± 6% yield.

**Fig. 2:**
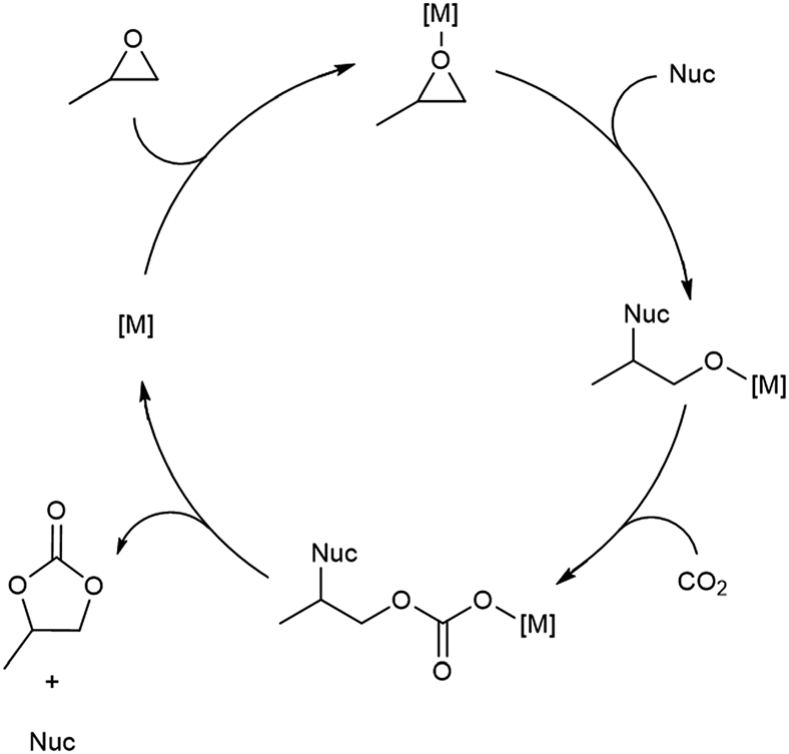
Proposed mechanism for conversion of PO to PC.

With flow conditions in hand, we first sought to eliminate the need for the TBABr co-catalyst in this system. This homogeneous co-catalyst is particularly disadvantageous in a flow configuration, because it must be added continuously along with the organic substrates. In addition, this additive could potentially obscure the inherent reactivity of the MOF catalysts.^25^ As shown in Fig. 2, the mechanistic role of the co-catalyst is to serve as a nucleophile to ring-open the epoxide once it is activated by coordination to an electrophilic metal center (presumably a metal in the node of the MOF).^31–33^ Importantly, previous work has shown that, in batch, the yield of PC without co-catalyst is low.^27^ However, we hypothesized that the high ratio of MOF catalyst to epoxide in a packed bed flow reactor relative to that in a batch reactor might result in increased reactivity, and that nucleophilic functional groups present at the MOF nodes (*e.g.*, hydroxides, chlorides, fluorides, or carboxylates derived from the MOF synthesis) and/or in solution (*e.g.*, water in the solvent) could potentially serve as nucleophiles under these conditions. Indeed, when the reaction was conducted under our standard flow conditions but without added TBABr, a 54 ± 2% single pass yield was obtained (Fig. 1). These co-catalyst free conditions were adopted moving forward for all subsequent studies.

### Impact of catalyst synthesis method and catalyst activation

**Fig. 3:**
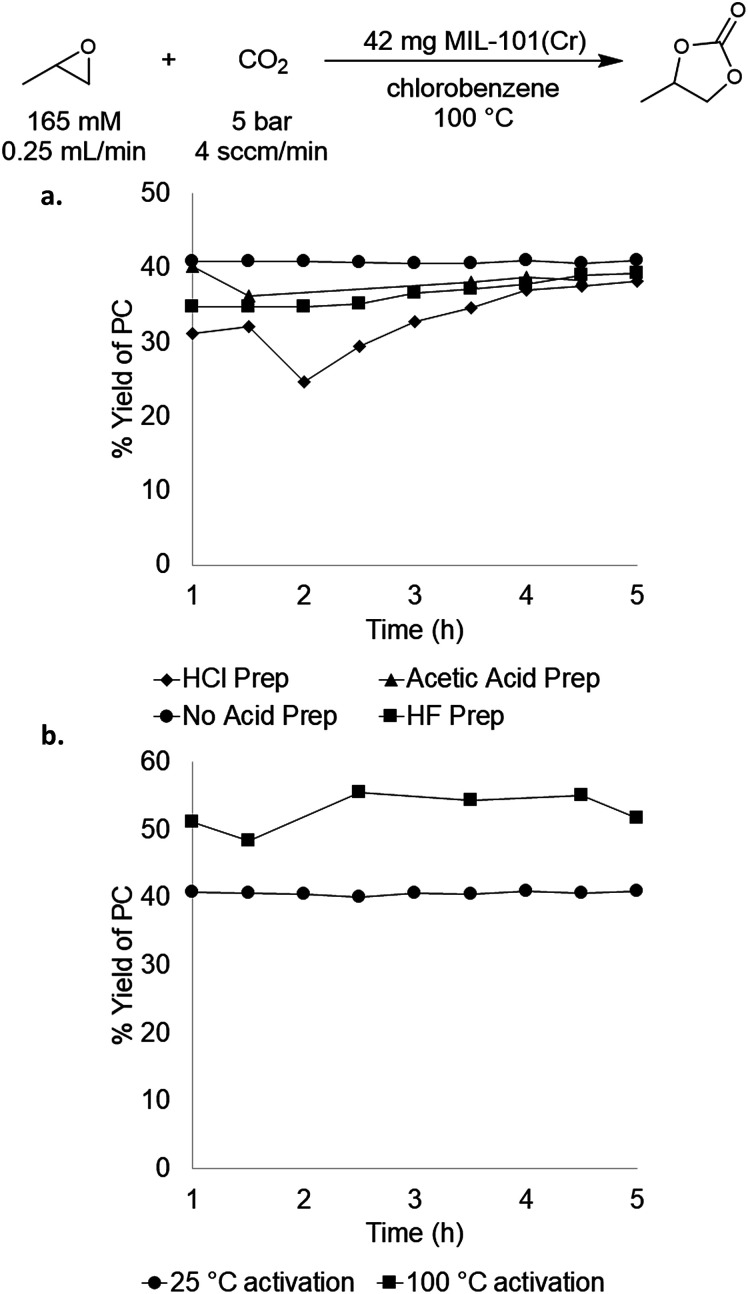
(a) Comparison of acids used for MOF synthesis; (b) comparison of 25 °C activation *versus* 100 °C activation of MIL-101(Cr).

We next sought to evaluate the impact of MOF synthesis method and activation procedure on the performance of MIL-101(Cr).^29,30,34^ MIL-101(Cr) has several reported preparations in the literature that vary primarily based on the acid utilized.^27,35–37^ The role of the acid during synthesis is not completely understood, but it is known that halides derived from the acid as well as hydroxides derived from water are incorporated into the framework during synthesis.^36^ As mentioned above, these halides or hydroxides could potentially act as nucleophiles during catalysis, thereby providing an endogenous co-catalyst. To test the impact of synthesis conditions on catalytic performance, MIL-101(Cr) was prepared using hydrochloric acid, hydrofluoric acid, and acetic acid as well as under acid-free conditions. In all cases, the other synthesis parameters (reaction time, temperature, metal salt, solvent quantity) and catalyst activation procedure were the same. As summarized in Fig. 3a, these four MIL-101(Cr) samples exhibited similar catalytic activity, with one-pass yields ranging from 33–41 ± 8%. These results indicate that the synthesis method has relatively minimal impact on catalyst performance in this system, and that catalysts containing different endogenous nucleophiles remain competent for PO carbonation.

We next probed the impact of MOF activation procedure on catalyst performance. MOFs are typically activated prior to catalysis by heating under vacuum in order to remove water/solvent that is in the MOF pores and bound to the metal nodes. We sought to assess whether this high temperature activation was beneficial or even necessary for PO carbonation catalysis. Initial experiments used MIL-101(Cr) that was activated according to the literature procedure (reduced pressure, overnight, 100 °C). This process is reported to yield MIL-101(Cr) with 2.47 mmol g^−1^ of active sites,^37^ which is close to our experimental value of 2.36 mmol g^−1^. The advantage of low temperature activation is that it minimizes the possibility of thermally-induced MOF decomposition, which is problematic for some Sc MOFs that we sought to compare to MIL-101(Cr) (*vide infra*). Room temperature activation yielded MIL-101(Cr) with 1.15 mmol g^−1^ active sites as determined by TPD. This suggests that this activation procedure does not remove all of the water/solvent molecules from the pores/metal nodes. Nonetheless, the room temperature-activated material maintained similar activity, affording 37 ± 4% steady state yield and a TOF of 19 h^−1^ (Fig. 3b). As such, the room temperature activation procedure was used for all of the studies below to enable direct comparisons with Sc-based materials that decompose at higher activation temperatures.

### Comparison of isostructural MOFs with different node metals

A key feature of metal organic framework catalysts is that they are highly modular. As such, the metal(s) in the nodes, the overall structure of the nodes, and the organic linker(s) can be systematically varied to tune catalytic performance. We next sought to exploit this tunability to generate second generation catalysts for PO carbonation. As shown in Fig. 2, epoxide carbonation involves Lewis acid activation of the epoxide,^37^ and the literature suggests that the Cr centers at the nodes of MIL-101(Cr) are the active sites in this system.^37^ Thus, we hypothesized that catalytic performance could be enhanced by increasing the oxophilicity of these sites.

Initial investigations focused on a MIL-101 series of isostructural MOFs synthesized with different metals at the nodes. In addition to MIL-101(Cr), analogous Fe- and Sc-based MOFs have been reported in the literature and have been shown to participate in Lewis acid-catalyzed reactions.^38^ Furthermore, a recent report by Kepp provided a quantitative scale of oxophilicity for these systems, with Sc = 0.8, Cr = 0.6, and Fe = 0.4 (higher numbers represent more oxophilic atoms).^39^

The MIL-101(Cr), (Fe), and (Sc) series was synthesized according to literature procedures^26–28^ and activated by several washes with ethanol followed by drying overnight under reduced pressure at 25 °C. Under our standard conditions, MIL-101(Cr) afforded a yield of 41 ± 1% at steady state operation and TOF of 21 h^−1^, for the material prepared under acid free conditions. In comparison, MIL-101(Fe) exhibited low activity, affording 1–2% yield under analogous conditions. This result is similar to the control reaction with no catalyst present, and is consistent with the lower oxophilicity of Fe.^33^ In contrast, the more oxophilic Sc-based catalyst, MIL-101(Sc), afforded higher activity than MIL-101(Cr) at initial time points. For instance, after 1 h the Sc and Cr MOFs afforded 53% and 41% yield of PC with TOFs of 87 h^−1^ and 21 h^−1^, respectively. However, in the case of MIL-101(Sc), subsequent time points revealed rapidly declining yields, culminating in <10% at 3 h. This result suggests that the MIL-101(Sc) catalyst is unstable under the reaction conditions. Indeed, PXRD analysis of the spent catalyst confirmed that MIL-101(Sc) loses crystallinity after 3 h under the reaction conditions. In contrast, minimal loss of crystallinity is observed for MIL-101(Cr) under analogous conditions. Overall, the high yield observed with MIL-101(Sc) at the start of the reaction provides promising initial evidence that Sc-based MOFs could offer improvements over the initial Cr-based catalyst.

**Fig. 4:**
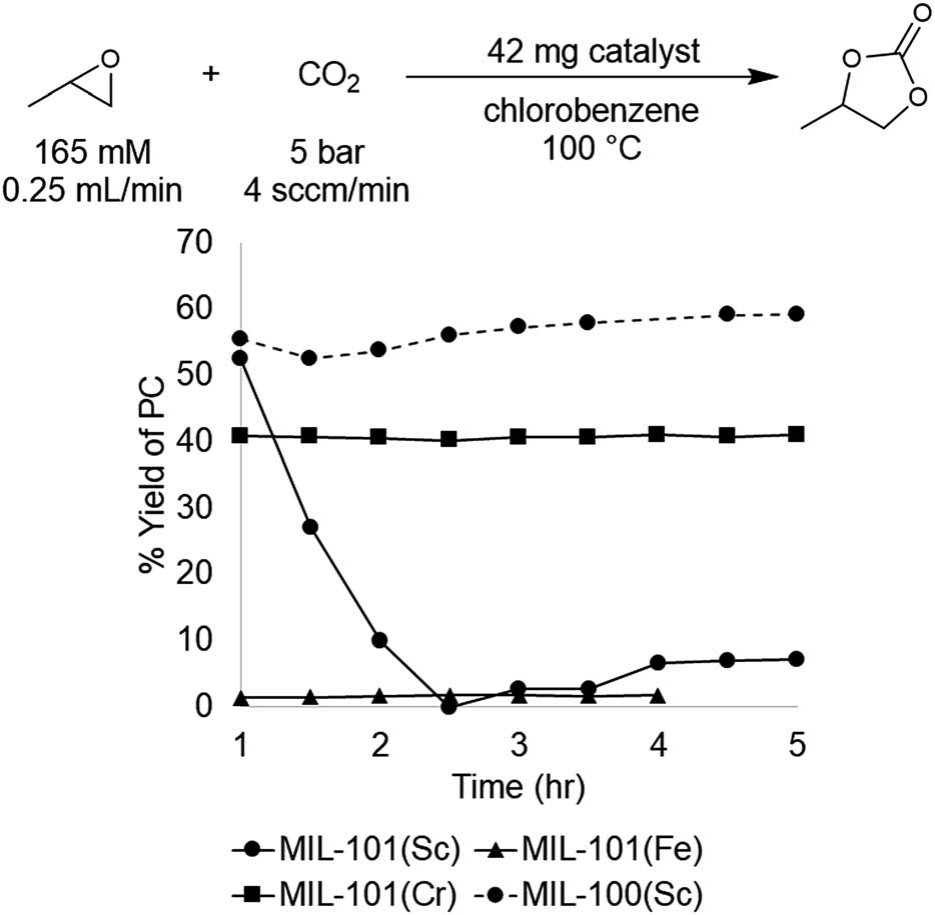
MOF node metal comparison for the conversion of PO to PC.

A recent report showed that MIL-101(Sc) has low thermal stability, rapidly losing crystallinity at temperatures >100 °C. In contrast, the related MOF, MIL-100(Sc), was reported to be stable up to 270 °C.^40^ The primary structural difference between the MIL-100 and MIL-101 series is the size of the pores and the pore windows. This size difference results from the tritopic trimesic acid used as the linker for MIL-100 *versus* the ditopic terephthalic acid linker used for MIL-101. However, the node geometry and overall superstructure is otherwise identical in both series, suggesting that MIL-100(Sc) could potentially maintain the activity of MIL-101(Sc) while exhibiting enhanced stability. Gratifyingly, the data show that MIL-100(Sc) affords the highest yield among all the investigated catalysts, with a product yield of 57 ± 5% and a TOF of 28 h^−1^ at steady state operation under the standard conditions (Fig. 4). Furthermore, this activity was maintained throughout the 5 h experiment.

### Comparison of MOFs with the same node metal

**Fig. 5:**
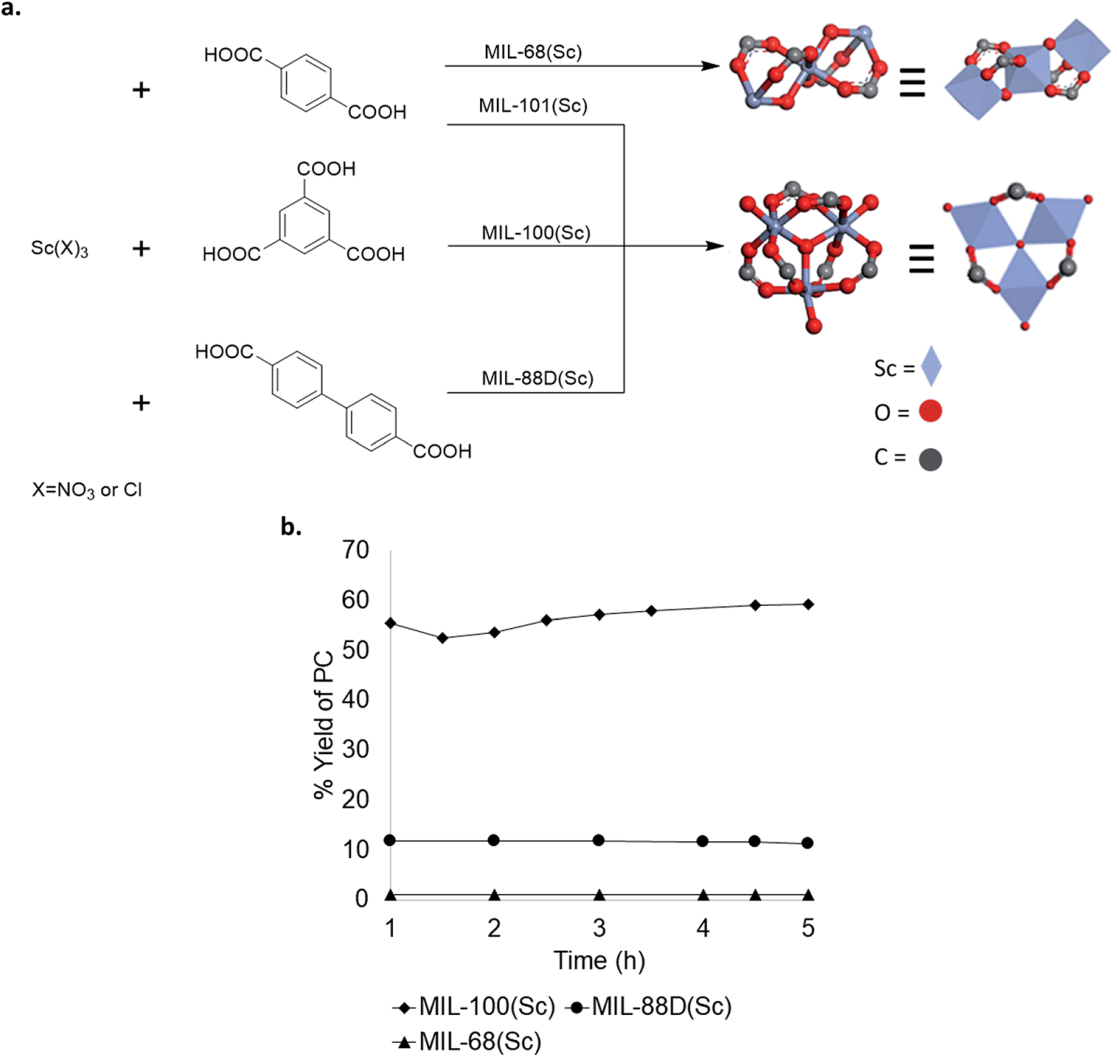
(a) Scandium-based materials derived from different metal node geometries; (b) comparison of Sc catalysts with different node geometries.

To further explore structure activity relationships in Sc-based MOFs, several analogues with different crystal structures and node geometries were explored (Fig. 5a). MIL-100(Sc) and MIL-88D(Sc) both possess the same node coordination environment, with one coordination site at each Sc^3+^ center occupied by a labile water molecule. In contrast, MIL-68(Sc) has a node coordination environment consisting of infinite chains of alternating Sc^3+^ and oxygen atoms, with the remaining coordination sites occupied by a poorly labile carboxylate ligand. As such, the Sc centers in MIL-68(Sc) are expected to be much less accessible for interaction with the epoxide. A comparison of catalytic performance in PO carbonation shows that MIL-100(Sc) and MIL-88D(Sc) afford 57 ± 5% and 11 ± 1% steady state yield and TOF 28 h^−1^ and 12 h^−1^, respectively. In contrast, MIL-68(Sc) affords <1% yield of PC (Fig. 5b). These results are consistent with the hypothesis that the presence of accessible Lewis acidic sites on the metal nodes is a key requirement for activity in these MOF-based catalysts. Collectively, these data provide guidance for the design of future generations of catalysts.

### Long term catalyst stability

The robustness and reactivity of a MOF is often dictated by its metal–ligand interactions.^41^ For example, some metal-containing clusters are susceptible to ligand substitution with water, leading to collapse of the frameworks upon exposure to moist environments.^42^ Other frameworks can collapse upon heating or even at room temperature.^28^ The stability of a MOF is a critical property that determines its practicality and potential in catalysis applications. Flow conditions provide an excellent platform for studying catalyst stability over long periods of time. The best catalyst, MIL-100(Sc), was subjected to 24 h of continuous operation at 100 °C in chlorobenzene. As shown in Fig. SI15,† MIL-100(Sc) exhibits minimal loss in reactivity over the time examined.

## Conclusions

In conclusion, a detailed evaluation of catalyst performance as a function of different variables for the carbonation of propylene oxide catalyzed by a variety of Cr, Sc, and Fe-based MOFs was performed. The effect of MOF synthesis yielded minor differences in MOF activity based on synthesis acid. As well, high temperature post-synthetic activation of MOF catalyst was shown to be beneficial but not necessary for significant reactivity. This systematic study yielded reaction conditions with significant advantages over previous protocols for this transformation. First, the requirement for TBABr as a co-catalyst has been eliminated. Second, MOF tunability has been leveraged to identify a Sc-based catalyst that outperforms the previously reported Cr material. Overall, these results provide important information on the parameters that impact MOF catalysis for the carbonation of propylene oxide. Future work will be focused on applying insights gained from these studies to the rational development of new MOF-catalyzed transformations.

## Conflicts of interest

There are no conflicts to declare.

## Supplementary Material

RA-008-C7RA13245J-s001
