# Supplementary material for: Structure activity relationships in metal–organic framework catalysts for the continuous flow synthesis of propylene carbonate from CO2 and propylene oxide
Source: RSC Adv. 2018 Jan 9;8(4):2132–7. doi: 10.1039/c7ra13245j (PMC9077212; doi:10.1039/c7ra13245j)
Supplement: RA-008-C7RA13245J-s001 [file RA-008-C7RA13245J-s001.pdf]

Electronic Supplementary Material (ESI) for RSC Advances.
This journal is © The Royal Society of Chemistry 2018

Supporting Information For:

Structure Activity Relationships in Metal-Organic Framework Catalysts for the Synthesis of Propylene Carbonate from CO_2 and Propylene Oxide

Bryant R. James, Jake A. Boissonnault, Antek G. Wong-Foy, Adam J. Matzger, and Melanie S. Sanford*

Department of Chemistry, University of Michigan, 930 N. University Avenue, Ann Arbor, MI 48109

Table of Contents:

I. **General Experimental**
II. **Synthesis and Characterization of Metal-Organic Frameworks**
III. **Design and Construction of a packed bed reactor**
IV. **Catalytic Carbonation Procedure**
V. **Sample GC Traces**
VI. **Catalyst Summary by Figure**
VII. **Additional Figures**

I. General Experimental

PXRD data were recorded at room temperature on a Bruker AXS D8 Advance powder diffractometer at 40 kV, 40 mA with a CuKα source (λ = 1.5406 Å) between 3 and 30° 2θ with a scan speed of 0.1 s/step and a step size of 0.04. Samples were measured on a glass microscope slide in an aluminum holder. All powder patterns were taken in a mixture (1:3 or 1:1) of MOF to diatomaceous earth. The diatomaceous earth is visible as a sharp peak at 22º 2Θ

TPD data were collected on a Micromeritics ASAP 2920, using a quartz reactor with a quartz wool bed according to the following procedure. The line was purged with He for 15 min (20 mL/min). NH_3 was passed through the sample for 180 min (20 mL/min) at 40 ºC in order to saturate the sites with NH_3. The gas was switched to He, and He was passed over the sample for 30 min at 40 ºC (30 mL/min) in order to remove physisorbed NH_3. The temperature was then ramped to 350 ºC (5 ºC/min) to desorb the chemisorbed NH_3, and the desorbed NH_3 was detected via mass spectrometry.

II. Synthesis and Characterization of MOFs

Synthesis of MOFs

MIL-101(Cr),[1] MIL-101(Fe),[2] MIL-101(Sc),[3] MIL-100(Sc),[3] MIL-88D(Sc),[3] and MIL-66(Sc)[3] were all prepared according to reported procedures. All reagents were obtained from either Fisher or Sigma-Aldrich and used without further purification, with the exception of N,N'-dimethylformamide (DMF) (which was dried over 4Å molecular sieves) and N,N'-diethylformamide (DEF) (which was purified by stirring over activated charcoal followed by filtration through silica gel).

MIL-101(Cr). $Cr(NO_3)_3 \cdot 9H_2O$ (400 mg, 1.10 mmol, 1 equiv), hydrofluoric acid (48-51% in H_2O, 1.10 mmol, 1 equiv), and terephthalic acid (76 mg, 0.46 mmol, 0.42 equiv) were combined in a Teflon-lined autoclave containing H_2O (4.8 mL). The resulting solution was placed in a room temperature oven and the temperature was ramped over 1 h to 220 ºC. The solution was heated at 220 °C for 8 h. The temperature ramp was found to be essential for the reproducible formation of active catalyst. The mixture was then cooled to room temperature, the solids were collected by centrifugation, and the product was washed with DMF (4 x 10 mL), water (3 x 10 mL), and ethanol (2 x 10 mL). The solids were dried under reduced pressure at either room temperature or 100 ºC for 16 h. Modifications to the synthesis of MIL-101(Cr) were performed by the substitution of HF with 1 equiv of HCl or acetic acid, as well as the omission of acid in the synthesis. The PXRD pattern for all samples matched that reported in the literature.[1, 4-6] TPD analysis of the material that was activated at room temperature yielded 1.2 mmol/g. TPD analysis of the material that was activated at 100 ºC yielded 2.4 mmol/g.

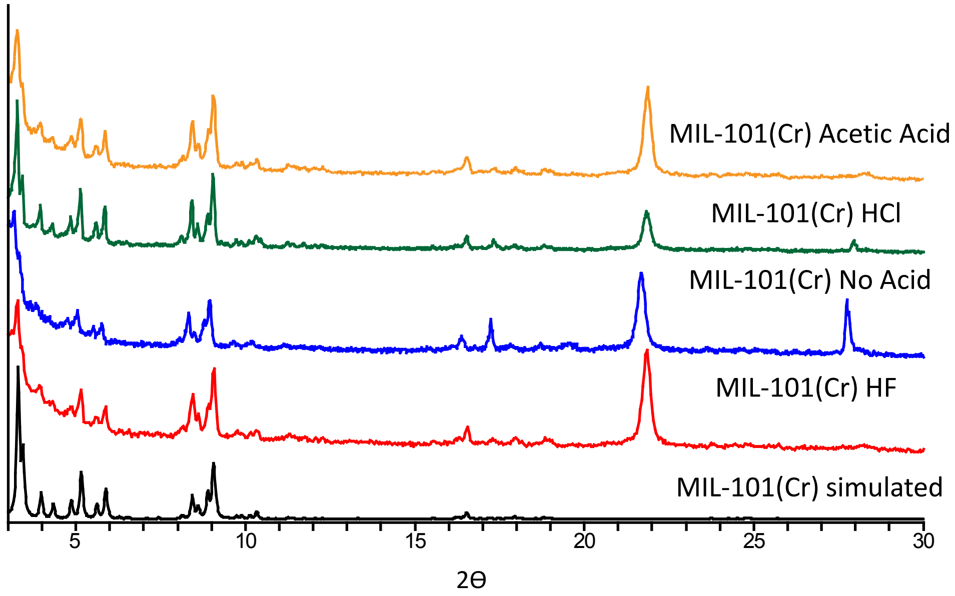

Figure S1. PXRD of MIL-101(Cr)

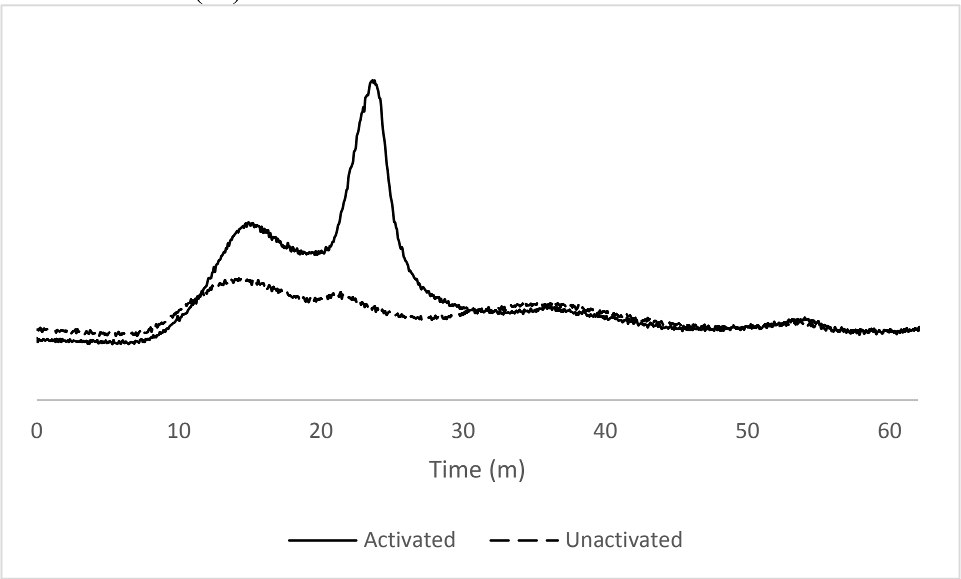

Figure S2. NH_3 TPD of MIL-101(Cr) no acid with activation at 125 ºC (activated) and activated at 25 ºC (unactived)

MIL-101(Fe). $FeCl_3$ (405 mg, 2.49 mmol, 1 equiv) and terephthalic acid (206 mg, 1.25 mmol, 0.5 equiv) were combined in a Teflon-lined autoclave containing DMF (15 mL) and H_2O (0.27 mL, 15 mmol, 6 equiv). This mixture was heated at 135 °C for 8 h. The mixture was cooled to room temperature, the solids were collected by centrifugation, and the product washed with DMF (3 x 10 mL) and ethanol (3 x 10 mL). The solids were dried under reduced pressure at room temperature for 16 h. The PXRD pattern for all samples matched that reported in the literature,[2] and TPD analysis yielded 1.3 mmol/g.

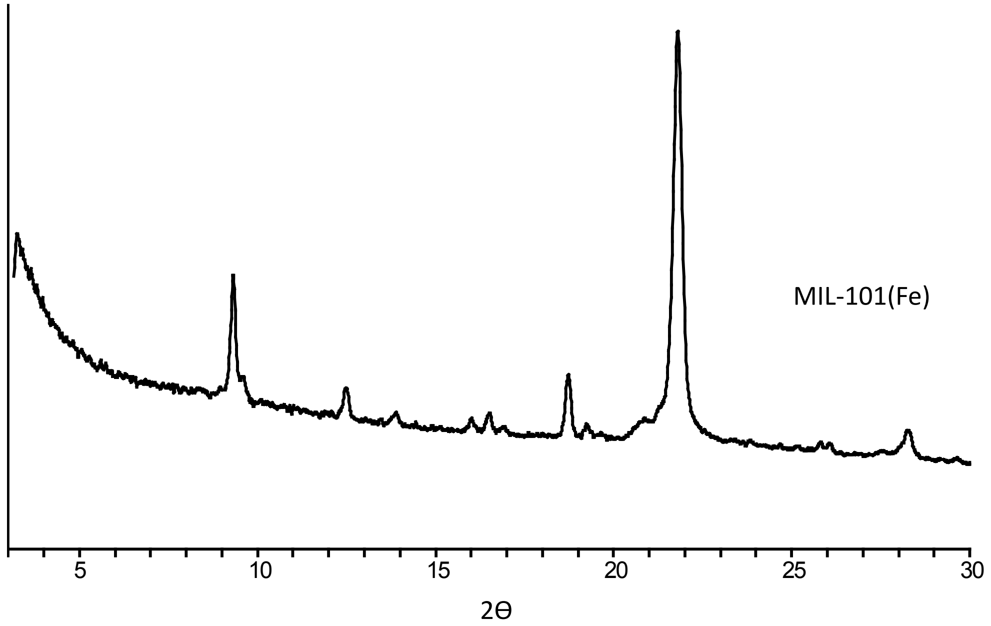

Figure S3. PXRD of MIL-101(Fe)

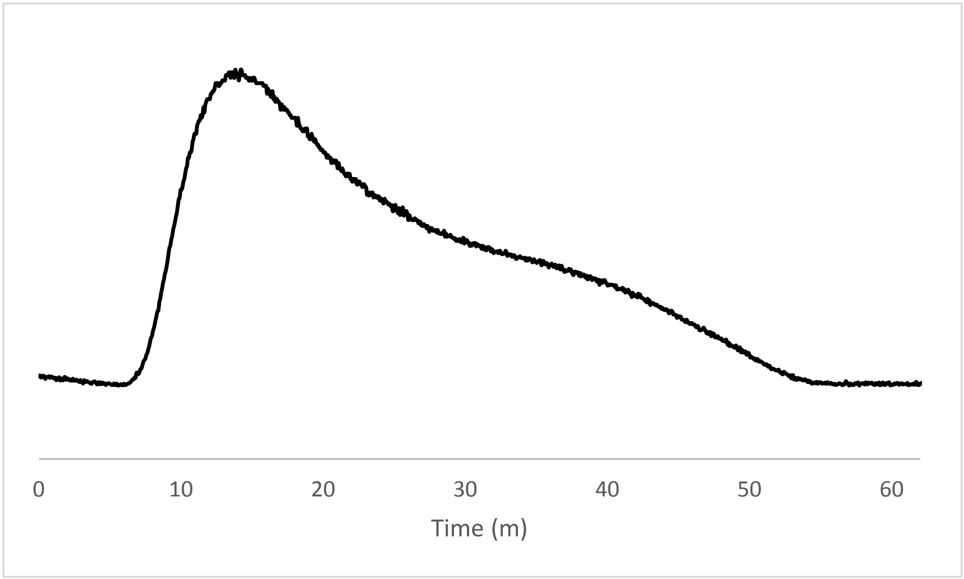

Figure S4. NH_3 TPD of MIL-101(Fe)

MIL-101(Sc). Solutions of $ScCl_3$ (0.580 mmol, 1.45 M in H_2O, 1 equiv) and terephthalic acid (106 mg, 0.638 mmol, 1.1 equiv) were combined in a Teflon-lined autoclave containing DMF (4 mL) and ethanol (200 proof, 5 mL). The autoclave was heated at 80 °C for 1 d. The mixture was cooled to room temperature and the solids were collected by centrifugation before being washed with DMF (3 x 10 mL) and ethanol (3 x 10 mL). The solids were dried under reduced pressure at room temperature for 16 h. The PXRD pattern for all samples matched that reported in the literature,[3] and TPD analysis yielded 0.4 mmol/g.

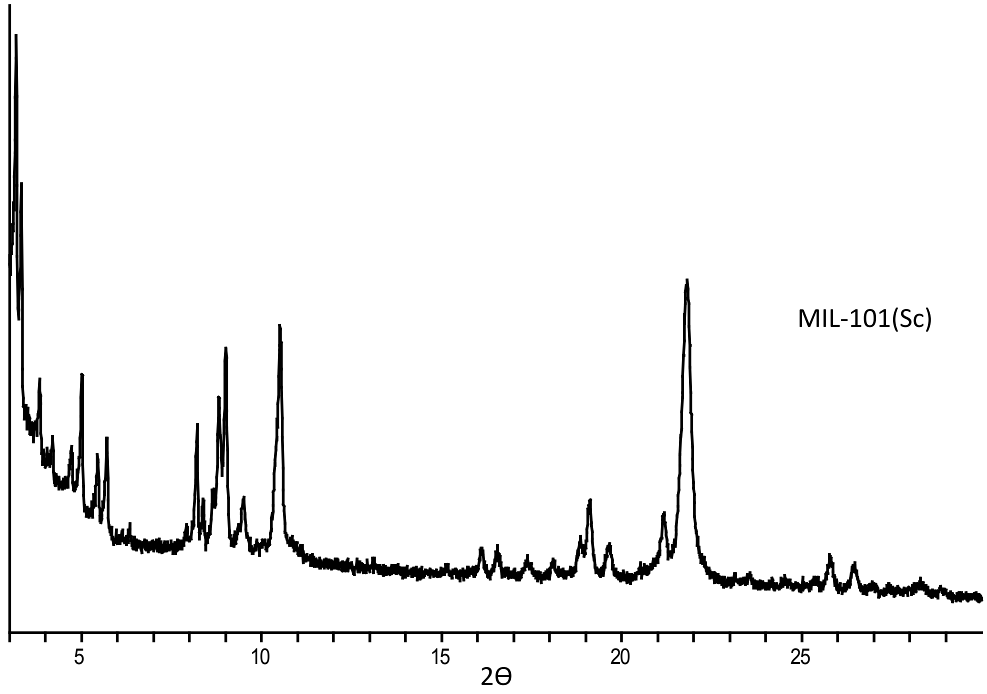

Figure S5. PXRD of MIL-101(Sc)

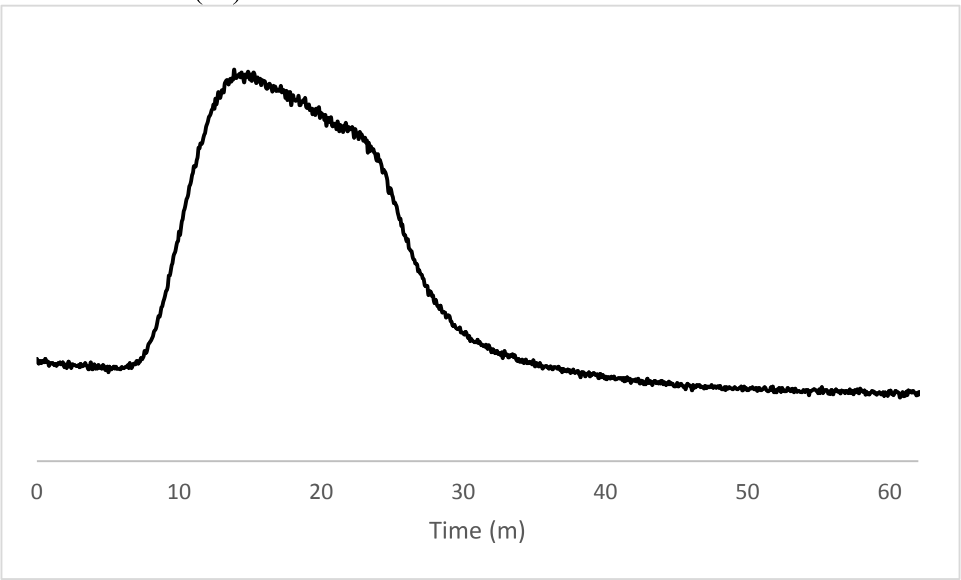

Figure S6. NH_3 TPD of MIL-101(Fe)

MIL-100(Sc). Benzene-1,3,5-tricarboxylic acid (90 mg, 0.428 mmol, 1 equiv) and scandium nitrate (246 mg, 1.07 mmol, 2.49 equiv) were combined in a Teflon-lined autoclave containing DMF (20 mL). The autoclave was heated at 150 ºC for 48 h. The mixture was cooled to room temperature and the solids were collected by centrifugation before being washed with water (3 x 10 mL) and ethanol (3 x 10 mL). The solids were then dried under reduced pressure at room temperature for 16 h. The PXRD pattern for all samples matched that reported in the literature,[3] and TPD analysis yielded 1.2 mmol/g.

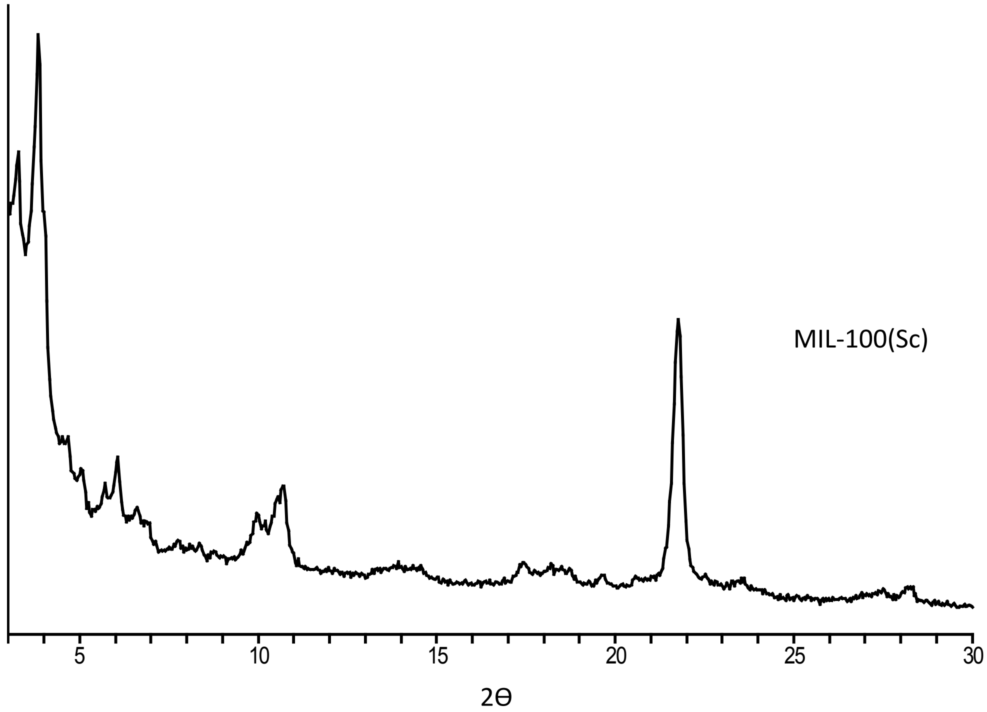

Figure S7. PXRD of MIL-100(Sc)

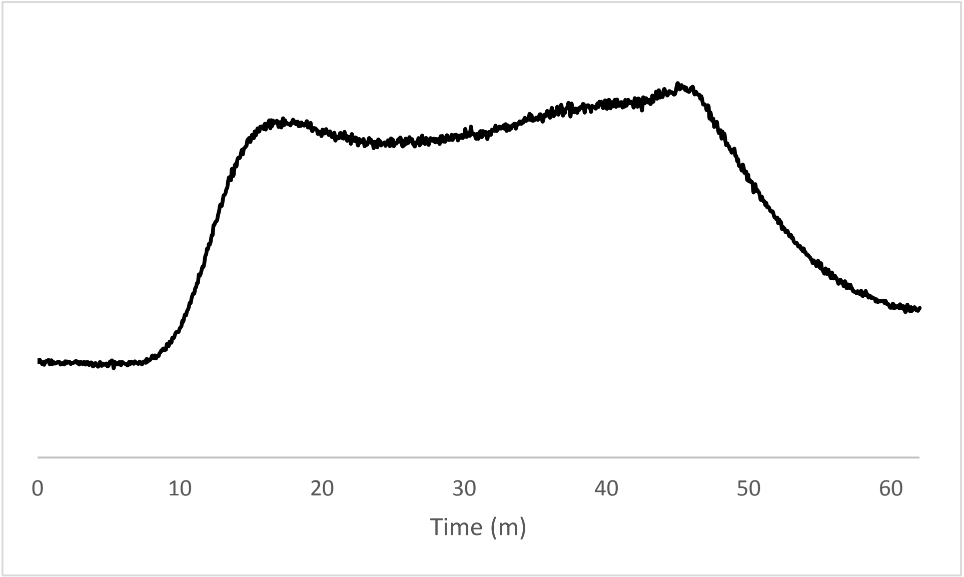

Figure S8. NH_3 TPD of MIL-100(Sc)

MIL-88D(Sc). Biphenyl-4,4'-dicarboxylic acid (121 mg, 0.500 mmol, 1 equiv) and scandium nitrate (143 mg, 0.535 mmol, 1 equiv) were combined in a Teflon-lined autoclave containing DEF (6 mL). This solution was heated at 110 °C for 72 h. The mixture was cooled to room temperature and the solids were collected by centrifugation before being washed with water (3 × 10 mL) and ethanol (3 × 10 mL) and dried in air at 60 °C for 16 h. The PXRD pattern for all samples matched that reported in the literature,[3] and TPD analysis yielded 0.5 mmol/g.

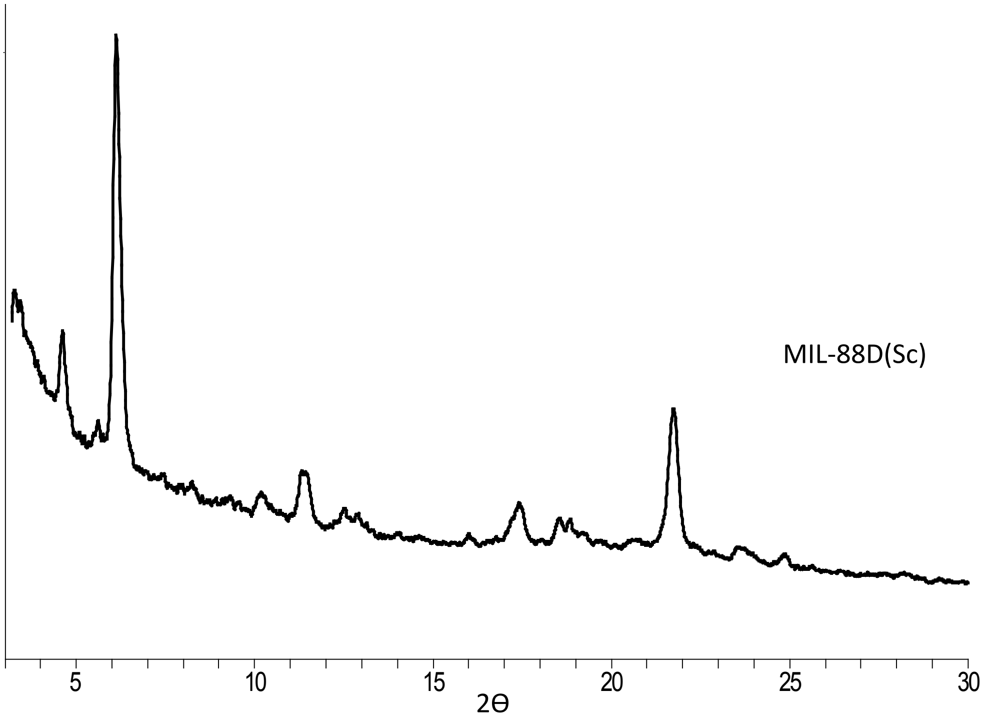

Figure S9. PXRD of MIL-88D(Sc)

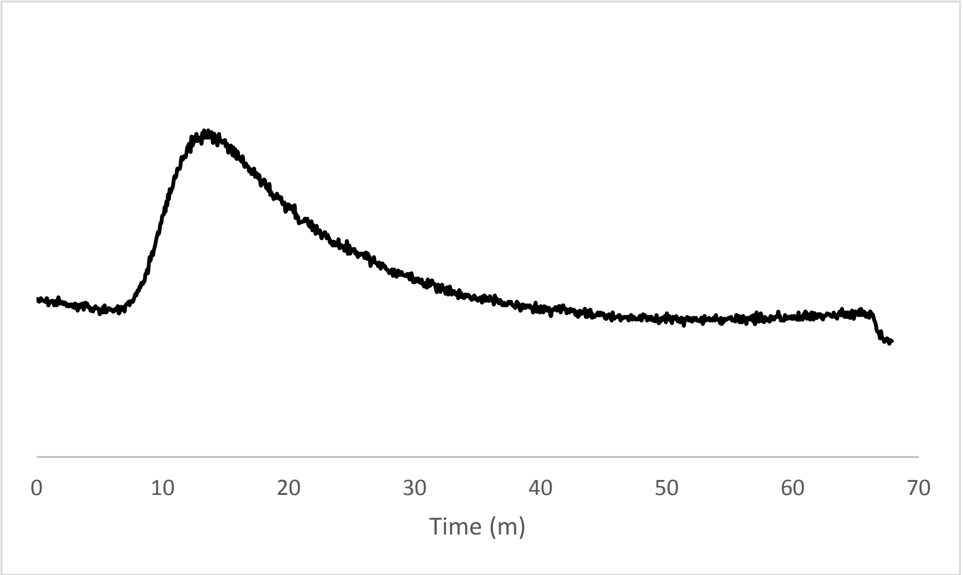

Figure S10. NH_3 TPD of MIL-88D(Sc)

MIL-68(Sc). Solutions of $ScCl_3$ (0.460 mmol, 1.45 M in H_2O, 1 equiv) and terephthalic acid (76 mg, 0.457 mmol, 1 equiv) were combined in a Teflon-lined autoclave containing DMF (3.0 mL), water (5.0 mL), and ethanol (200 proof, 5 mL). This mixture was heated at 90 ºC for 12 h. The resulting solids were collected by centrifugation, washed with ethanol (3 × 10 mL), and dried in air at 60 °C for 16 h. The PXRD pattern for all samples matched that reported in the literature,[3] and TPD analysis yielded 0.6 mmol/g.

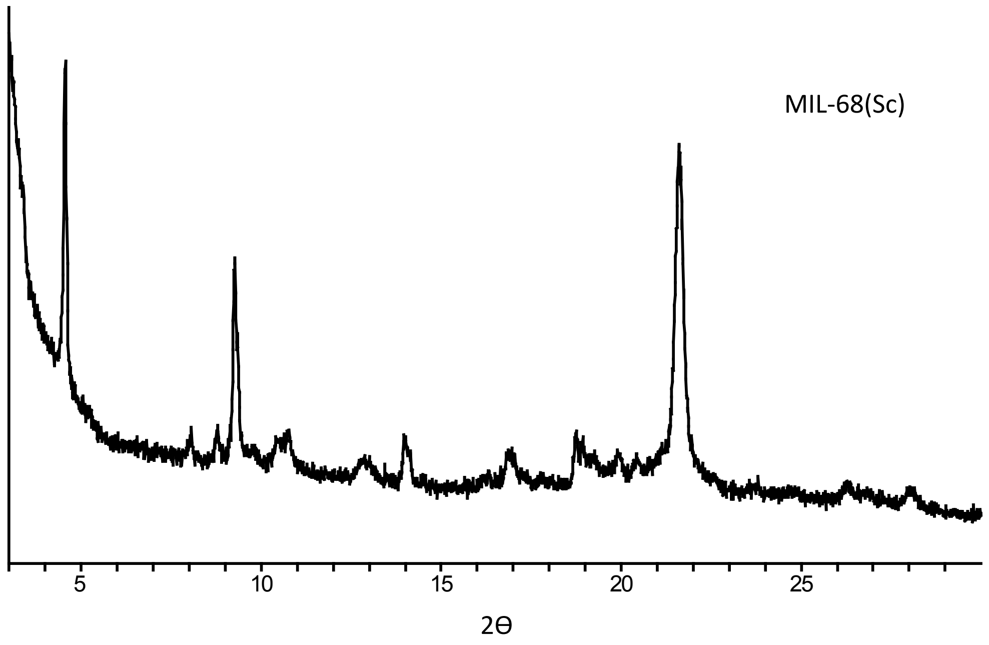

Figure S11. PXRD of MIL-68(Sc)

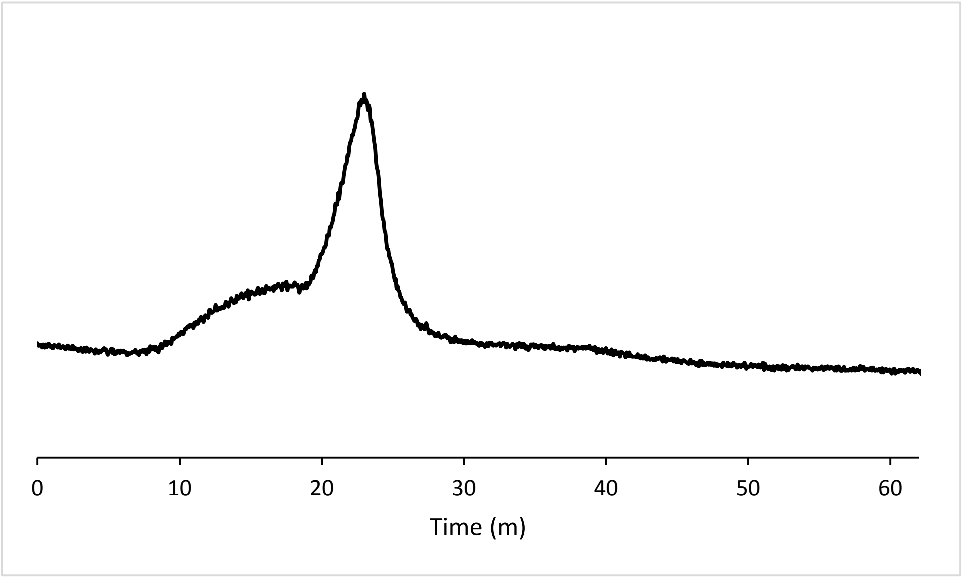

Figure S12. NH_3 TPD of MIL-68(Sc)

III. Design and construction of a packed bed reactor

An HPLC pump was used to deliver liquid stock solutions, and a gas tank and high-pressure mass flow controller were used to deliver high-pressure carbon dioxide. A four-way dynamic mixer was used to allow for thorough mixing and expansion if more stock feeds were needed. The oven was constructed from copper pipe covered in silicone-wrapped heating tape connected to a temperature controller. The catalyst bed was a glass tube fitted with Vespel/graphite ferrules and packed with a mixture of catalyst and diatomaceous earth. A thermocouple was placed in direct contact with the outside surface of the bed for temperature regulation. An ice bath was used to minimize evaporation as the solution exits the reactor. The system pressure was controlled using a back-pressure regulator, and the outlet consisted of a three-way ball valve to facilitate sampling (**Figure S13)**.

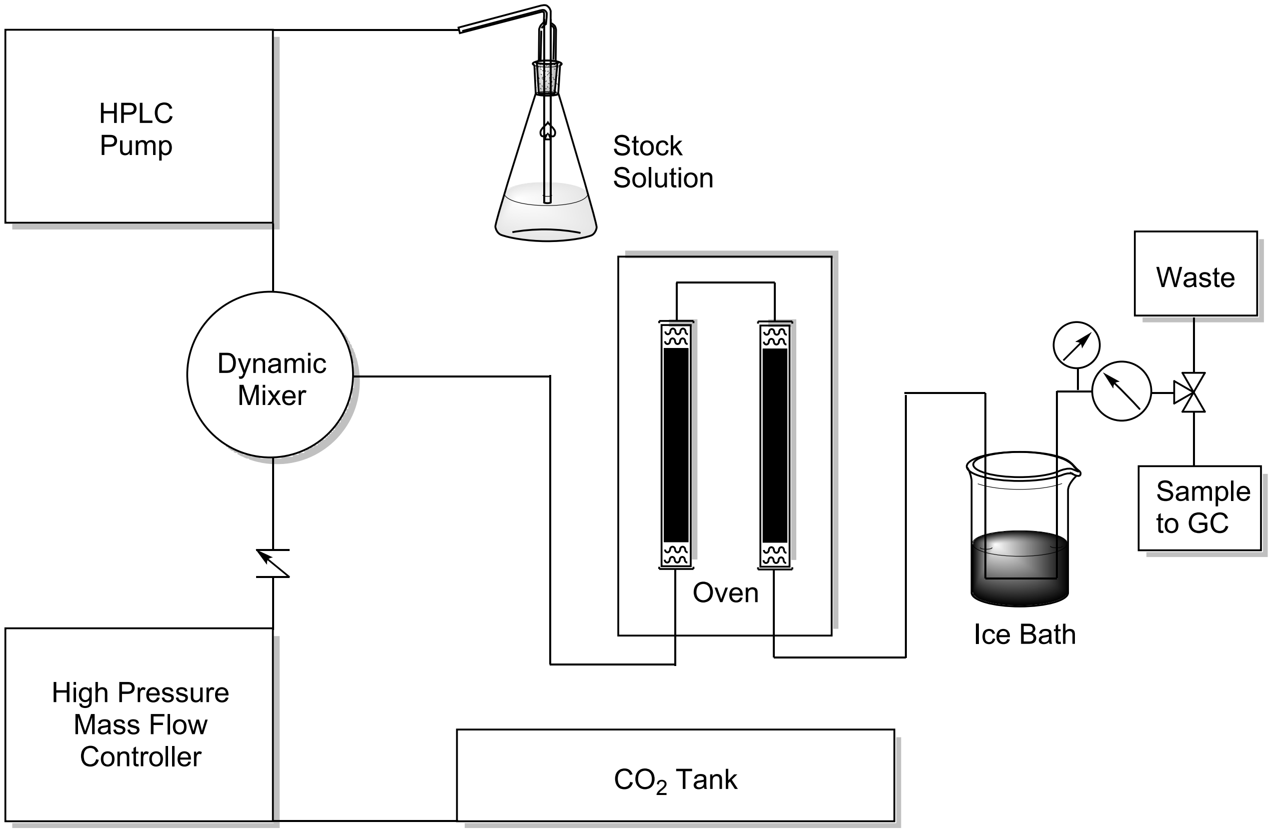

Figure S13. Packed-bed flow reactor

IV. Catalytic Carbonation Procedure. The catalyst and Fisher lab-grade diatomaceous earth were combined in either a 1:1 w/w ratio or a 1:3 w/w ratio. The mixture was transferred to a mortar and pestle and ground until visibly homogeneous. The mixture was packed between two glass wool plugs in a ¼ inch OD, 1/20-inch wall thickness glass tube. The column was installed into the flow system, and both gas and stock solution streams were started simultaneously. CO_2 flow rates were varied from 1-4 sccm/min, and stock flow rates were varied from 0.25-0.5 mL/min. A second column in series was designed into the system for larger quantities of catalyst. The second column was packed with glass wool when smaller quantities of catalyst were used. The system was allowed to run until reaching the desired system pressure (between 1 and 10 bar) before heating was started. Aliquots were collected every 30-60 min and analyzed by GC-FID on a Shimadzu GC-17A. Mesitylene was included in the feed as internal standard.

V. Sample GC traces

Peaks correspond to propylene oxide (1.2 min), Mesitylene (6.9 min) and propylene carbonate (7.2-7.7 min). Longer GC experiments, up to 30 min, show no additional peaks.

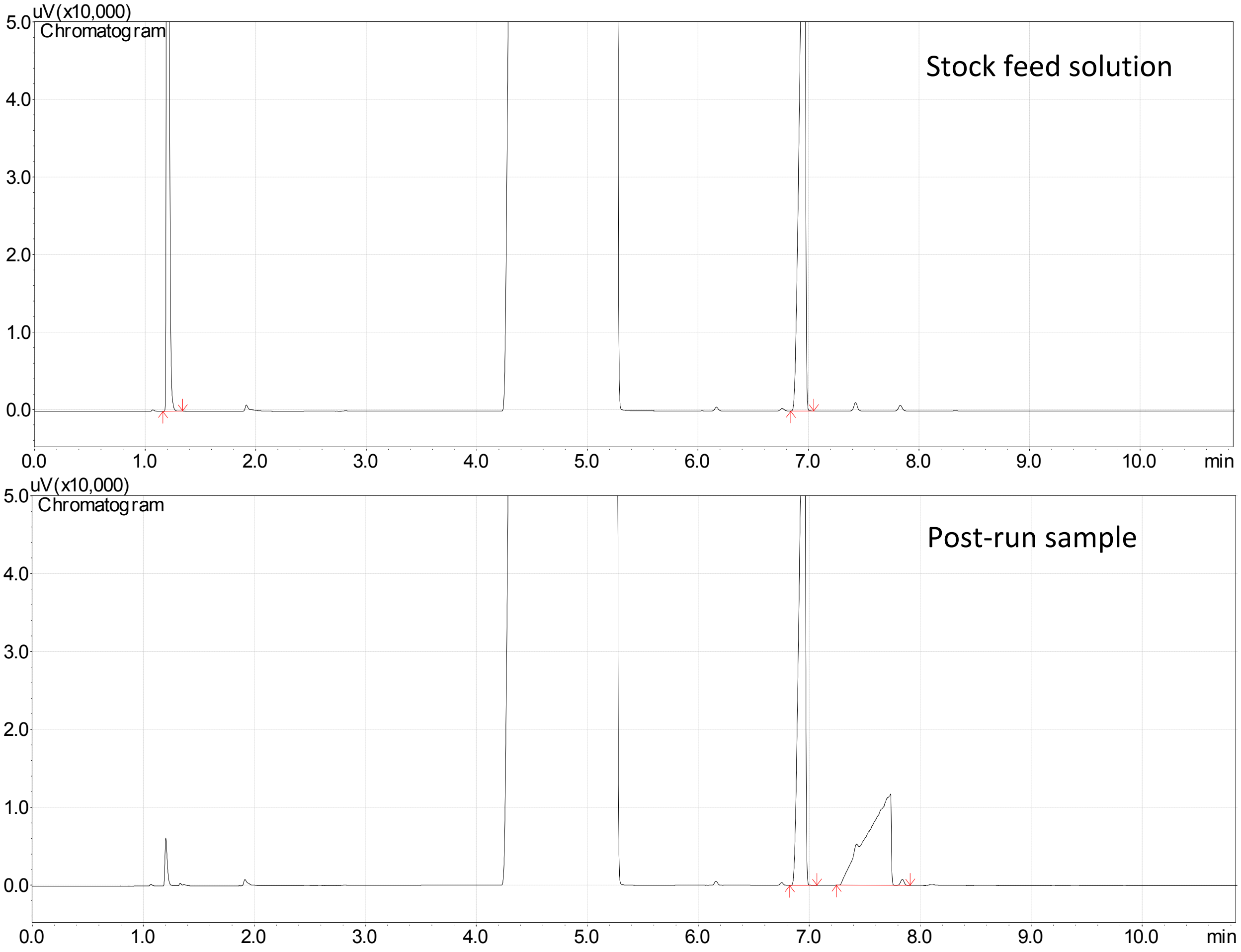

VI. Catalyst Summary by Figure

For **Figure 1**, MIL-101(Cr) was the sole catalyst used. The catalyst was synthesized using hydrofluoric acid and activated at 100 ºC under vacuum for 16 h. The activated catalyst was combined in a mortar and pestle with an equal weight of diatomaceous earth and 82 mg of this mixture was loaded into a column.

For **Figure 3a**, MIL-101(Cr) was the catalyst used and varies only in synthesis method. The catalyst was synthesized using hydrofluoric acid, hydrochloric acid, acetic acid or no acid and activated at either 25 ºC under vacuum for 16 h. The activated catalyst was combined in a mortar and pestle with an equal weight of diatomaceous earth and 82 mg of this mixture was loaded into a column.

For **Figure 3b**, MIL-101(Cr) was the sole catalyst used. The catalyst was synthesized using hydrofluoric acid and activated at either 100 ºC or 25 ºC under vacuum for 16 h. The activated catalyst was combined in a mortar and pestle with an equal weight of diatomaceous earth and 82 mg of this mixture was loaded into a column.

For **Figure 4**, MIL-101(Sc), MIL-101(Fe) and MIL-100(Sc) were synthesized as stated above and activated at 25 ºC under vacuum pressure for 16 h. MIL-101(Cr) was synthesized using acid-free conditions and activated at 25 ºC under vacuum for 16 h. The activated catalyst was combined in a mortar and pestle with an equal weight of diatomaceous earth and 82 mg of this mixture was loaded into a column.

For **Figure 5b**, MIL-100(Sc), MIL-88D(Sc) and MIL-68(Sc) were synthesized as stated above and activated at 25 ºC under vacuum for 16 h. The activated catalyst was combined in a mortar and pestle with an equal weight of diatomaceous earth and 82 mg of this mixture was loaded into a column.

For **Figure S14**, MIL-100(Sc) was synthesized as stated above and activated at 25 ºC under vacuum for 16 h. The activated catalyst was combined in a mortar and pestle with an equal weight of diatomaceous earth and 82 mg of this mixture was loaded into a column.

VII. Additional Figures

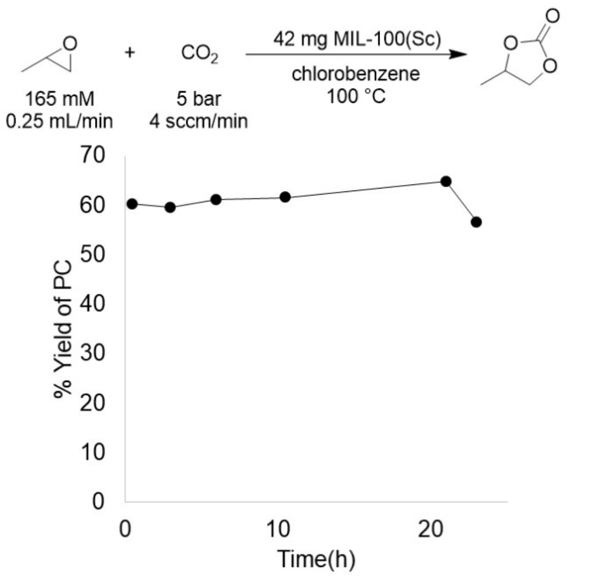

Figure S14. MOF node metal comparison for the conversion of PO to PC synthesis

References

1. G. Ferey, C. Mellot-Draznieks, C. Serre, F. Millange, J. Dutour, S. Surble and I. Margiolaki, *Science*, 2005, **309**, 2040-2042.
2. A. Santiago-Portillo, S. Navalón, F. G. Cirujano, F. X. L. i. Xamena, M. Alvaro and H. Garcia, *ACS Catalysis*, 2015, **5**, 3216-3224.
3. L. Mitchell, B. Gonzalez-Santiago, J. P. S. Mowat, M. E. Gunn, P. Williamson, N. Acerbi, M. L. Clarke and P. A. Wright, *Catal. Sci. Technol.*, 2013, **3**, 606-617.
4. P. B. S. Rallapalli, M. C. Raj, S. Senthilkumar, R. S. Somani and H. C. Bajaj, *Environ. Prog. & Sustain. Energy*, 2015
5. L. Bromberg, Y. Diao, H. Wu, S. A. Speakman and T. A. Hatton, *Chem. Mater.*, 2012, **24**, 1664-1675.
6. D.-Y. Hong, Y. K. Hwang, C. Serre, G. Férey and J.-S. Chang, *Adv. Funct. Mater.*, 2009, **19**, 1537-1552.
